# Robotic gaze and human views: A systematic exploration of robotic gaze aversion and its effects on human behaviors and attitudes

**DOI:** 10.3389/frobt.2023.1062714

**Published:** 2023-04-10

**Authors:** Michael Koller, Astrid Weiss, Matthias Hirschmanner, Markus Vincze

**Affiliations:** ^1^ Automation and Control Institute, TU Wien, Vienna, Austria; ^2^ Human Computer Interaction Group, TU Wien, Vienna, Austria

**Keywords:** human-robot interaction, gaze aversion, mutual gaze, eye-tracking, user experience

## Abstract

Similar to human–human interaction (HHI), gaze is an important modality in conversational human–robot interaction (HRI) settings. Previously, human-inspired gaze parameters have been used to implement gaze behavior for humanoid robots in conversational settings and improve user experience (UX). Other robotic gaze implementations disregard social aspects of gaze behavior and pursue a technical goal (e.g., face tracking). However, it is unclear how deviating from human-inspired gaze parameters affects the UX. In this study, we use eye-tracking, interaction duration, and self-reported attitudinal measures to study the impact of non-human inspired gaze timings on the UX of the participants in a conversational setting. We show the results for systematically varying the gaze aversion ratio (GAR) of a humanoid robot over a broad parameter range from almost always gazing at the human conversation partner to almost always averting the gaze. The main results reveal that on a behavioral level, a low GAR leads to shorter interaction durations and that human participants change their GAR to mimic the robot. However, they do not copy the robotic gaze behavior strictly. Additionally, in the lowest gaze aversion setting, participants do not gaze back as much as expected, which indicates a user aversion to the robot gaze behavior. However, participants do not report different attitudes toward the robot for different GARs during the interaction. In summary, the urge of humans in conversational settings with a humanoid robot to adapt to the perceived GAR is stronger than the urge of intimacy regulation through gaze aversion, and a high mutual gaze is not always a sign of high comfort, as suggested earlier. This result can be used as a justification to deviate from human-inspired gaze parameters when necessary for specific robot behavior implementations.

## 1 Introduction

In human–robot interaction (HRI) settings, such as visual joint attention tasks or human–robot conversations, both robotic and human gazes guide the attention of the interaction partner, influence the attitude toward them, and provide and shape the rhythm of the interaction. Therefore, how robot engineers integrate robotic gaze into an HRI setting is an impactful design decision.

Gaze behavior is specified by low-level parameters, such as animation curves of head and eye movements, specific gaze targets or directions, and frequency and duration of fixations. Similarly, gaze depends on the current context, such as the interaction partner (Kendon, 1967). Previous studies have reported a relationship between one high-level gaze behavior parameter, namely, the gaze aversion ratio (GAR), and the perceived user experience (UX) (Zhang et al., 2017). A positive UX is central to accepting social robots in our everyday lives (Weiss et al., 2009). In current studies with social humanoid robots such as Pepper,^1^ the GAR for a positive UX is often determined through a human-inspired approach (Andrist et al., 2014): gaze timings in human–human interaction (HHI) are recorded and replicated in robotic systems.

We consider GAR a central parameter in conversational HRI settings because it is a good representation of the overall gaze behavior of a robot. Even if the robot behavior designer does not explicitly model the behavior around a chosen GAR, the GAR of a behavior can always be computed. Gaze aversion is also a social interaction parameter for which a broad body of HRI work exists (Admoni and Scassellati, 2017). A common approach is to create a human-inspired gaze behavior. Thus, deviating from such human-inspired gaze parameters produces relevant complementary findings to the human-inspired approach.

Additionally, deviating from human gaze behavior is necessary if the robotic gaze has specific additional goals and limitations. This includes movement restrictions imposed by the robot embodiment or sensor limitations that impose minimum observation times for detecting certain objects (Ban et al., 2017) if the robot’s gaze coincides with the camera view. This problem is relevant if design principles of honest anthropomorphism are followed, for example, if the camera is installed in the head of the robot in its assumed gaze direction (Kaminski et al., 2016).

The effects of deviating from such HHI-inspired GAR have not yet been systematically studied, and there are unexplored assumptions: (1) Is the human-inspired parameter setting optimal for a specific robot embodiment and scenario? Other parameter settings might be equally or more appropriate for a specific robot embodiment, and these will never be identified when adhering to human-inspired parameters as closely as possible. (2) Assuming the HHI parameter setting is optimal, how much does a deviation from that optimum degrade the UX? Insights into these questions benefit robot designers who need to balance the social and technical aspects of robotic gaze behavior.

In this study, we explore a broad range of values for the robotic GAR for the Pepper robot and whether there are systematic effects on the different dependent interaction measures. We chose this parameter because it is a high-level gaze behavior descriptor and can be derived for every robotic social gaze behavior. Thus, we argue that the findings of this study are relevant to many different experimental settings. There are different implications for future robot- and human-centered research, depending on the outcome. If only one high-performing setting can be interpreted as “close to the human behavior,” it might be reasonable to only inspect settings close to this behavior in the future. Otherwise, if we observe several high-performing settings or negligible differences, robot designers will have more freedom in their choices. For technology-focused approaches, if there is knowledge about more lenient acceptable gaze timings, it is easier for robot designers to approach the range of acceptable parameters in their implementation.

We created an experimental design to determine the GAR that enables a positive UX without being derived from HHI. As measures for UX, we chose the feeling of being attended, the feeling of comfort within the interaction, and the perceived interaction capabilities of the robot (Pfeiffer et al., 2011). Furthermore, we recorded the gaze behavior of the participants as an implicit measure of UX because human gaze behavior, including gaze aversion (Chen and Clarke, 2017), is closely linked to affect and emotion. We additionally measure other interaction parameters, namely, the interaction duration and word count uttered by the human participants, as these variables have been linked to affective, emotional, and attentional states (Burra and Kerzel, 2021).

In this study, we present the following contributions:

1. We introduce an experimental design to vary one parameter of robot behavior, namely, the GAR, in a wide range of possible values for a specific anthropomorphic robot, namely, Pepper, without assuming that human behavior is optimal.
2. We implement a minimal-animacy robot behavior that isolates the effect of the mere ratio of gazing at the human conversation partner *versus* averting the gaze to the side.
3. We conducted a user study (*n* = 101) in which we recorded, evaluated, and interpreted a rich dataset (10.48436/frswc-4dn44) composed of gaze-tracking records over the whole duration of the interaction, other behavioral measures, such as spoken word count and interaction duration, and self-reported attitudes toward a social robot.
4. We discuss guidelines for implementing robot behavior that adheres less strictly to human-derived parameters.

The remainder of this work is organized as follows: related work about gaze and eye-tracking in previous HRI experiments is presented in Section 2. In Section 3, the research questions are posed, followed by the used methods in Section 4. Then, results are presented in Section 5, and discussed and transferred into guidelines in Section 6. We finish with a conclusion in Section 7.

## 2 Related work

In this section, we first present related work in both human and robotic gaze behavior research. We review different forms of social gaze and why it is a crucial nonverbal social modality for humans. Then, we review how social gaze behavior has been implemented in previous HRI studies with different experimental designs. After that, we review relevant user studies incorporating eye-tracking devices and different ways of extracting empirical findings from eye gaze data. Human eye gaze reveals latent variables, such as attributions toward the robot. These measures have been used in HHI and HRI studies.

### 2.1 Robotic and human gaze behavior in HRI

The GAR is a high-level descriptor of gaze. However, gaze aversion can occur in different circumstances with varying goals. There are findings concerning the neural correlates of gaze behavior in humans and monkeys (Perrett et al., 1985). Different groups of cells in the macaque temporal cortex were fully activated when the monkey was confronted with different views of the head. In Emery (2000), gaze as a social cue is discussed as an evolutionary phenomenon that allows humans to interpret complex social scenarios in the following taxonomy: (1) mutual gaze describes a setting where two interaction partners are looking at each other’s faces. (2) Averted gaze occurs when one partner looks away from the other. (3) Gaze following occurs when one partner notices the averted gaze of the other and then follows their line of sight to a point in space. (4) Joint attention is similar to gaze following, except that the person averting the gaze has a specific object as the gaze target, and the other person attends to that object. (5) Shared attention is a bidirectional process where both partners simultaneously perform a mix of mutual gaze and joint attention on the same object. Thus, both know that the other is looking at the target object. (6) Theory of mind (ToM) uses higher-order cognitive strategies and performs a mental state attribution to the interaction partner. Thus, one partner can determine that the other intends to interact with an object or react to a stimulus because they intend to achieve a goal by doing so or have a particular belief about it that leads to a specific action.

This classification does not separate gaze aversion into a single category. Gaze aversion can occur in the averted gaze, as well as gaze following, joint attention, and shared attention. However, the GAR remains an objective, condensed descriptor of gaze behavior.

Like above, the GAR can differ in the context of its social function and occur through different gaze acts: five functions of social gaze and six types of gaze acts have been identified (Srinivasan and Murphy, 2011). The five functions are establishing social agency (reinforcing presence and aliveness), communicating social attention (showing interest in humans), regulating the interaction process (managing participation and turn-taking), manifesting interaction content (looking at the object of interest), and projecting mental state (expressing emotions and intentions). The six gaze acts—fixation (at target object or person), short glance, gaze aversion (away from a person), concurrence (repetitive horizontal or vertical movement to interrupt fixations), confusion (signified by consecutive rapid gaze shifts back and forth), and scan (several short glances to random points in space)—have been identified.

Specifically for conversational settings, gaze aversion has additional functions (Andrist et al., 2014): floor management, intimacy regulation, and indication of cognitive effort. Floor management gaze behavior consists of gaze aversions during speech pauses to indicate that the conversational floor is being held and an interruption of the speaker is not desired. Intimacy regulation between two conversation partners is achieved by different degrees of gaze aversion, depending on the relationship of the conversation partners, the conversation topic, and scenario properties, such as the physical distance of the two speakers. Cognitive effort can lead to more gaze aversions of the speaker, as they can better focus on the planning and delivery of the following utterances.

We summarize that gaze aversion behavior is highly dependent on the conversation setting, the two interaction partners, and dynamic aspects during the flow of conversation. However, there have been attempts to derive empirical average percentages for gaze at the interaction partner and mutual gaze: Argyle (1994) and Argyle et al. (1994) reported that one person in a conversational dyad spends approximately 60% of the time looking at the conversation partner. 30% of the interaction time is spent in mutual gaze. While listening, people gaze more often at the conversation partner (71%) than while speaking (41%). The authors report high interpersonal variance for gaze aversion. In our experimental setting, the robot takes on the role of the listener in a dyadic conversational setting. Thus, A GAR value of 0.3 (corresponding to gazing at the partner about 70% of the time) constitutes the HHI standard.

Rightly, previous work focused on determining robotic gaze behavior that improves the interaction (Admoni and Scassellati, 2017, Chapter 5). However, the complexity of the implementation and other test-theoretic design demands would have led to infeasibly large sample size for more fine-grained experimental setups. Thus, in the different test conditions, the presumed high-performing implementation is often compared against a deliberately poorly performing or neutral condition. This type of research question is valid for verifying specific implementations. Our work aims to answer the complementary question of which insights can be generated for future robot behavior designs when there is no explicit split between different performance groups. This is also motivated by (Admoni and Scassellati, 2017, p. 37): “It is tempting to assume that perfectly matching robot gaze behaviors to human gaze behaviors will elicit identical responses from people, but this is not always the case,” as was shown by Admoni et al. (2011).

### 2.2 Eye-tracking in HRI

Eye-tracking in HRI research produces detailed and rich data samples during interactions and provides task-specific sensory data to robotic learning algorithms. Including eye tracking as a dependent variable in experimental designs studying conversational HRI settings is a common technique and has yielded several insights into the connection between gaze behavior and UX.

Perugia et al. (2021) operationalized eye-tracking measures for the feelings and attitudes of participants over time toward different robot embodiments in a conversational HRI scenario. They found that human gaze aversion is an indicator of the uncanniness of a robot. Similarly, the more a participant gazed at the robot in a joint task, the worse they performed. Gaze aversion in a social chat is an indicator of the uncanniness of a robot. Liking of the robot and mutual gaze develops congruently throughout multiple interactions. Specifically, the reported uncanniness decreased, whereas mutual gaze by the participants increased. Moreover, the authors argued that later interactions represent a more stable gaze pattern after the novelty effect wore out.

Additionally, in order to pure conversational settings, mutual gaze has also been used to estimate the social engagement of participants in joint task settings (Castellano et al., 2010; Papadopoulos et al., 2016).

Sidner et al. (2005) interpreted the freely chosen interaction duration of their participants with a social robot as an implicit measure of interest in engagement with the robot. The amount of looking at the robot is a measure of attention toward the robot. They found that in both experimental conditions (talking/gesturing robot) of a conversational object reference game, participants spent about 70% of the time looking at the robot in both conditions with no differences in the self-reported likability of the robot.

Yu et al. (2012) employed a data collection procedure including video recording and eye-tracking of human participants in an HRI scenario, where participants taught object names to either a robot or another confederate participant. They reported differences in the amount of time spent gazing at the face of another human or a robot interaction partner. Intimacy regulation (Argyle and Dean, 1965) in a conversation is achieved by averting the gaze from the other person. Thus, inanimate characters such as persons on TV and arguably social robots who exhibit limited sociability receive more gaze than physically present human interaction partners. This result is an indication against the human-inspired gaze control design approach.

Baxter et al. (2014) interpreted the direction and timing of a human’s gaze over time toward a robot while interacting by comparing different gaze metrics derived from temporally split interaction thirds. They noticed a decrease in gaze at the robot’s head over time and thus proposed this measure as a proxy for engagement in the interaction and the perceived social agency of the robot.

The human eye gaze acts as an implicit measure of engagement (Jokinen, 2018) to determine whether an anthropomorphic conversational robot is gazed upon as a communicative agent or a technological tool. The robot performed the task of reading a newspaper article out loud. Participants exhibited a high level of gaze toward the face of the robot. This is regarded as evidence for the communicative agent hypothesis, confirming the media equation hypothesis (Reeves and Nass, 1996). There was a dynamic change in gaze targets: at the beginning of an interaction, there is more gaze toward the head of the robot than at the end, although the small sample size limits the validity of this observation.

Eye gaze behavior is also linked to persistent personality traits (Ijuin and Jokinen, 2020). Analyses of HHI and HRI conversations revealed that participants gaze more at the partner’s body in HRI than in HHI and more at the partner’s face in HHI than in HRI, and there is a positive correlation between the character trait openness and the average duration of gazing toward partner’s body in HRI. This interpersonal effect is modulated by the situational effect of intimacy regulation during a conversation.

Sabyruly et al. (2015) reported a temporal effect in their conversational HRI setting; people who spend more time with the robot have less favorable attitudes toward it. Participants spent, on average, 50% of the time looking at the face of the robot, with large interpersonal differences. They confirmed previous findings that the maximal pupil diameter correlates with cognitive load and concluded that telling a story constitutes a measurable cognitive load.

Zhang et al. (2017) reported that a prolonged stare of a robot at the participants increased their arousal. In their interactive gaze experimental condition, a prolonged mutual gaze of the participant at the robot correlated positively with a higher rating in fluency, fun, and connectedness.

Similarly, Broz et al. (2012) found in HHI conversations that gaze aversions and mutual gaze highly depend on both interaction partners. They reported a positive correlation between mutual gaze and the combined person’s agreeableness, as well as their familiarity. Concerning gaze dynamics between the two interaction partners, they reported correlations implying that mutual gaze cannot be increased by only one of the two participants by simply looking at their opposite for a more extended amount of time.

In summary, eye-tracking has several favorable properties for HRI research: it produces more objective data than self-report measures. The resulting data are richer and more granular than the questionnaire results. Eye-tracking data reveal behavior, attitudes, and emotional states that escape the conscious verbalization of participants when filling out questionnaires. Previous studies allow us to link gaze behavior with emotional states and attitudes.

## 3 Research questions

As our work aimed to determine whether viable parameter ranges for HRI behavior implementations can be found without adhering to previously determined HHI parameters, we varied one gaze behavior factor, namely, the GAR. It is defined as the ratio of time averting the gaze from the interaction partner to the gaze cycle time. A GAR of 1.0 relates to always averting the gaze from the interaction partner, whereas a GAR of 0.0 relates to always gazing at the interaction partner.

Different robot behaviors can implement the same GAR as different movement profiles and gaze cycle lengths can average the same amount of time gazing at and away from the interaction partner. In our study, we wanted to establish whether the mere ratio of time spent gazing at the interaction partner’s face and the time spent averting the gaze is already a significant factor for how the users experience the behavior of the robot without varying different dynamics, such as animation curves.

The Softbank Pepper^2^ robot was chosen as the robot embodiment. The humanoid shape allows a natural implementation of gaze aversion, whereas the un-actuated face of the robot (except for LED lit eyes) does not signal a false affordance to participants; that is, participants do not expect elaborate facial animation during the interaction. Pepper robots are currently a widespread social robotics research platform, and thus findings can be incorporated into future research for the same platform (and similar platforms like the Softbank NAO robot^3^). Lastly, these robot platforms adhere to the honest anthropomorphism design principle; that is, the robot only gathers visual information in a visual field where the anthropomorphic head is pointed. Designing robot behavior for robot embodiments that do not violate the users’ trust poses additional challenges but is necessary for a trustworthy relationship.

Our primary research interest was to determine to what degree the GAR influences the behavior and attitudes of human interaction partners. To operationalize this, we derived research questions that can be grouped into three categories: 1) the gaze behavior of participants, 2) interaction behavior, and 3) attitudes.

1) Gaze behavior:

• **RQ 1a:** Does the GAR affect the fixation durations of the participants?
• **RQ 1b:** Does the effect of the GAR on the fixation durations change during the interaction?
• **RQ 1c:** Does the GAR influence the fixation sequence behavior of the participants?

2) Interaction behavior:

• **RQ 2a:** Does the GAR affect the number of words spoken by the participants?
• **RQ 2b:** Does the GAR affect the interaction duration?

3) Attitudes:

• **RQ 3a:** Does the GAR affect participants’ perception of the robot’s attention toward them?
• **RQ 3b:** Does the GAR affect participants’ feeling of comfort during interaction with the robot?
• **RQ 3c:** Does the GAR affect participants’ perception of the robot’s interaction capabilities?

## 4 Methods

We designed an experiment in a conversational interaction setting and validated it in a pilot study (Koller et al., 2019). During the interaction, the robot greets a human participant and asks about their favorite movie. Then, it listens to their statement. When the human stops talking, the robot thanks the participant and says goodbye to conclude the interaction.

**FIGURE 1:**
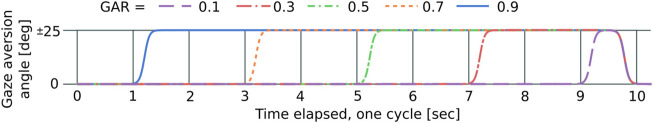
Timing of gaze focus transitions for the five conditions. At 0°, Pepper looks at the human. The movement time of 0.5 s is included in the gaze aversion time. At 9.5 s, all GAR conditions return to 0°. Pepper’s different gaze angles are −25°, 0°, and +25°.

The gaze aversion of the robot was the main behavior of the robot while the participant was talking. It was manipulated as the independent variable. The gaze aversion behavior cycled every 10 s. During a cycle, the robot acted according to a specific GAR and a predetermined gaze dynamic. Figure 1 shows the operationalization of the independent variable GAR across five conditions (0.1, 0.3, 0.5, 0.7, and 0.9).

We added additional animacy behavior as gaze aversion head shifts occur only every few seconds to avoid the robot appearing as “turned off” during the listening phase of an experiment trial. Johnston and Thomas (1981) reported twelve animation principles derived from animation practices in animated movies. These principles have also found use in the animation of robots (Ribeiro and Paiva, 2019) to improve aspects such as readability or likeability. There are several typically used animation profiles in HRI experiments to convey animacy. Mota et al. (2016) implemented the following idle movements on a Baxter robot in an HRI scenario: eye blinking and gazing, opening and closing grippers, and arm movements. For our experimental design, we iteratively arrived at the following animacy behavior: eye blinking behavior through Pepper’s LED eyes, “breathing” behavior through subtle body movements, and short and randomized head movements added to the explicit gaze shifts. Moreover, homogeneous interaction length among the participants was desirable for the experiment. We conducted several HHI pre-trials where a human played the role of the robot. The participants rated 2–3 min as a comfortable interaction length.

During the greeting and farewell part of the interaction, the robot performed several utterances and gestures (see Supplementary Material). If a participant stopped talking for more than 3 s, the robot made an utterance to ask for more input from the user (“Was that all?”) without an additional gesture. The experimenter triggered the beginning of the experiment, that is, the robot greeting, the robot utterance after a human speaking pause, and the robot farewell utterance manually in the Wizard-of-Oz (WoZ) style (Riek, 2012). In a pilot study (Koller et al., 2019), we conducted the main experiment without eye-tracking and only three GAR scenarios 0.1, 0.5, and 0.9 (*n* = 10). These trials were used for wizarding training, and the first author acted as the only wizard for all experiments. The wizard knew the research questions of the experiment, but there were no hypotheses about group comparisons that were likely to be significant. With respect to the guidelines elaborated by Fraser and Gilbert (1991), the only wizard recognition and production variables were detecting speech pauses of 3 s and then triggering the following phase of the experiment. If the duration was less than 2 min, the robot asked, “Was that all?” to encourage the participants to talk longer. When a 3 s pause occurred after 2 min or after the robot had already asked to elaborate once, the farewell utterance was triggered.

The microphone integrated into the Pepper robot was not reliable enough for the automatic detection of human speech. In other HRI experiments, this difficulty was solved by providing a hand-held or head-mounted external microphone to the human participant (Hirschmanner et al., 2021). In our experiment, however, this would have made it too cumbersome for the participants, as they already wore a head-mounted eye-tracker. Information about the degree of wizarding was given to the participants only after completing the questionnaire. Riek (2012) argues that a precise specification of the scenario for the participants is also important. This was achieved by providing written instruction of the experiment before consenting and another verbal instruction provided by the experimenter. Concerning the eye-tracking procedure, the following steps were performed: 1) explanation of the eye-tracking hardware, 2) fitting of the eye-tracker, 3) adjusting the eye-tracking camera for robust pupil detection, 4) performing the single marker calibration choreography, 5) confirming eye-tracking quality, and 6) recording. Then, the robot started the interaction with a greeting, which the experimenter triggered (see Supplementary Material). The experiment design and data processing procedure were peer-reviewed by the TU Wien Pilot Research Ethics Committee.

For the main experiment, all participants (*n* = 101) were recruited from the premises of TU Wien on the fly, most of whom were students. The participants were asked to give written consent after reading a short introduction to the experiment. After the experiment, each participant received 10 € as compensation. We performed the experiment in a room of the TU Wien library. The room was only illuminated with artificial ceiling lights and the window shades were closed to achieve consistent lighting conditions. There was no sound except the fans of the robot and the computer the experimenter used.

**FIGURE 2:**
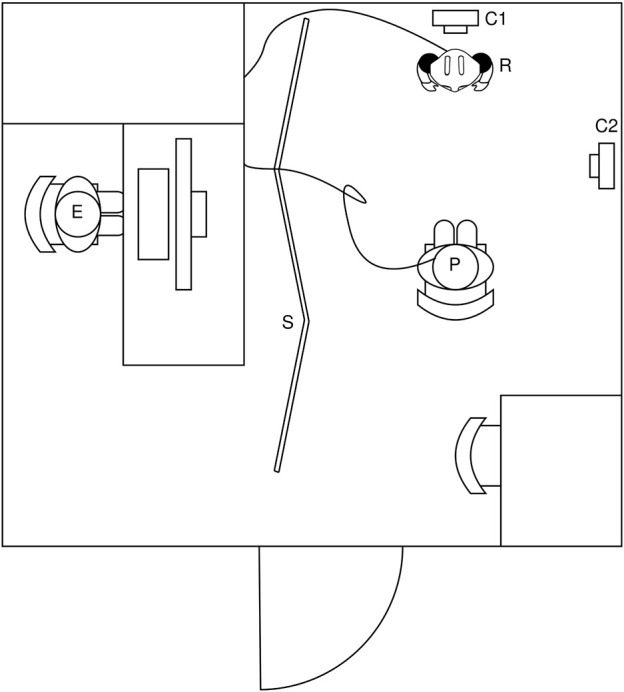
Schematic layout of the experiment room (4 m × 3.5 m). Participant with eye tracker (P), experimenter (E), robot (R), cameras (C1, C2), and room separator (S).

The room was split into two sides by an opaque room separator (Figure 2). On one side, the participant sat on a chair in front of the Pepper robot at a distance of 110 cm between the chair and the base of the robot. Two cameras were positioned near the robot and the participant. On the other side of the separator, the experimenter executed the robot interaction script from a desktop. The WoZ controller could see the camera feed of the pepper robot and listen to the participant.

COVID-19 hygiene measures were taken in all sessions. Pencils and the eye-tracking device were disinfected before every trial, and the room was ventilated between trials. The experimenter wore an FFP2/N95 mask at all times, whereas the participants were asked to remove their masks to accommodate the eye-tracker.

To measure behavioral differences between GAR conditions, we record the eye gaze behavior, the interaction duration, and the word count during the interaction as objective measures. To measure the attitudes of the participants toward the robot, we compiled sections from validated questionnaire series, namely, Godspeed (Bartneck et al., 2009) and BEHAVE-II (Joosse et al., 2013). We chose scales that are appropriate for the interaction setting and research questions. Each section consists of four five-point Likert-type scales, each with two positively and two negatively connoted terms, with an additional “I don’t know” option. The aggregated scale of attention consisted of the ratings responsive, interactive, ignorant, and unconscious. The aggregated scale of comfort consisted of the ratings creepy, feeling nervous, warm, and pleasant. The aggregated scale of interaction capabilities consisted of the ratings artificial, incompetent, intelligent, and sensible. To avoid misunderstandings, we provided the participants with a glossary for the terms of the questionnaire (see Supplementary Material for all forms used during the experiment).

Additionally, we posed three open-ended interview questions after participants completed the questionnaire to detect problems during the experiment and get qualitative impressions: “How did it feel to talk to the robot?”, “Do you have additional thoughts about the robot?”, and “Do you have any other thoughts about the experiment?”

The resulting dataset is securely and confidentially stored at the TU Wien research data repository (10.48436/frswc-4dn44).

## 5 Results

The data analysis is performed for human gaze behavior (i.e., gaze fixations on a specific region of interest (ROI) and pupil measures), interaction behavior (i.e., word count and duration), and attitudinal measures (i.e., self-reported measures). The human gaze data are analyzed in different ways: 1) aggregated statistical measures of the whole interaction, 2) aggregated statistical measures for each interaction, 3) human gaze sequences, and 4) temporal correlation of robot and human gaze shifts.

### 5.1 Data preparation and descriptive statistics

A total of 101 participants took part in the experiment, of whom four participants had to be excluded due to technical problems with the experimental procedure and 1 participant due to misunderstanding the instructions, resulting in a sample size of 96 for the self-reported measures and interaction behavior measures of word count and duration (*n* = 96, age: mean = 24.26, SD = 4.02). A total of 43 participants identified as female and 53 as male participants. Every participant reported daily computer use. Regarding the experience with robots, the participants reported interaction with a robot as follows: never (61), once (12), or a few times (23). No one interacted with robots on a regular basis until the time of the experiment.

For the analysis, the negatively formulated scales ignorant, unconscious, creepy, nervous, artificial, and incompetent were inverted. Items answered with “I don’t know” were treated as missing values and occurred with the following frequencies: responsive, 1; interactive, 2; ignorant, 4; unconscious, 5; creepy, 0; feeling nervous, 0; warm, 0; pleasant, 0; artificial, 0; incompetent, 5; intelligent, 6; and sensible, 3.

The aggregated scale of attention is composed of the items responsive, interactive, ignorant (inverted), and unconscious (inverted). The composite scale of comfort is composed of the items creepy (inverted), nervous (inverted), warm, and pleasant. The composite scale of capability is composed of the items artificial (inverted), incompetent (inverted), intelligent, and sensible. Cronbach’s alpha tests for the aggregated scales of attention (*α* = 0.84), comfort (*α* = 0.62), and interaction capabilities (*α* = 0.73) were performed.

For the attitudinal and behavioral measure tests, descriptive statistics per test condition are presented at the top of Table 1. Discrepancies in the group sizes arose because we also tried to have similar group sizes for valid eye-tracking data, where additional errors occurred that led to the exclusion of some participants.

**TABLE 1:** Top: descriptive statistics of participants (*n* =96) for attitudinal and interaction behavior measures. Bottom: descriptive statistics of participants (*n* =88) for gaze-related measures.

GAR	0.1	0.3	0.5	0.7	0.9
*N*	19	19	21	17	20
Age mean (SD)	26.7 (6.4)	23.4 (2.6)	23.6 (2.5)	22.8 (2.2)	24.3 (3.8)
Gender	9 f/10 m	8 f/11 m	10 f/11 m	8 f/9 m	8 f/12 m
*N*	19	17	19	16	17
Age mean (SD)	26.7 (6.4)	23.4 (2.6)	23.4 (2.4)	23.1 (2.5)	24.6 (3.8)
Gender	9 f/10 m	6 f/11 m	9 f/10 m	7 f/9 m	6 f/11 m

For the eye-tracking analysis, from the 96 participants who completed the self-report questionnaire, eight more needed to be excluded due to poor average pupil detection confidence (<0.6) (*n* = 88, age: mean = 24.22 years, SD = 4.07, gender: 51 m, 37 f) (bottom of Table 1).

**FIGURE 3:**
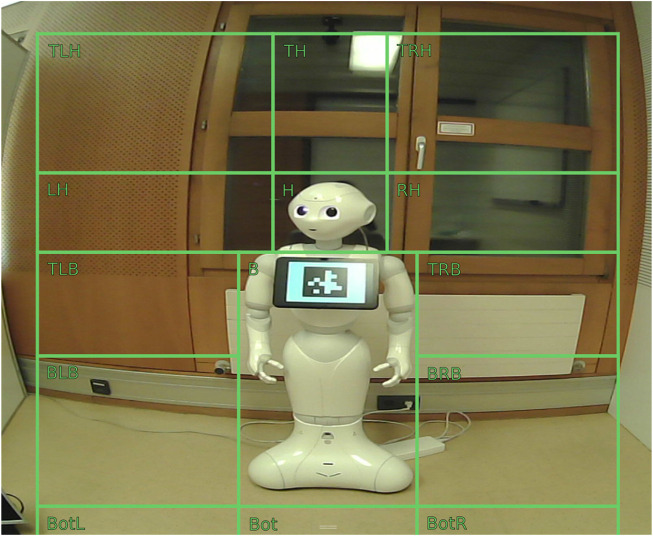
Regions of interest (ROI) relative to the Pepper robot as seen from the world view camera of the eye-tracking device from top left to bottom right: top left head (TLH), top head (TH), top right head (TRH), left head (LH), head (H), right head (RH), top left body (TLB), body (B), top right body (TRB), bottom left body (BLB), bottom right body (BRB), bottom left (BotL), bottom (Bot), and bottom right (BotR).

Gaze data during the interaction period were recorded with a Pupil Labs Core eye-tracking device. The gaze data were aggregated as follows: from the gaze data per recorded frame, fixations were detected through the Pupil Labs fixation detection algorithm.^4^ Thus, each fixation is described by a duration and a gaze coordinate, which can be mapped into world space and, more specifically, onto a specific ROI in the world space. On the most granular level, the following ROIs are defined, as depicted in Figure 3: robot head (H), robot body (B), top left of head (TLH), top of head (TH), top right of head (TRH), left of head (LH), right of head (RH), top left of body (TLB), top right of body (TRB), bottom left of body (BLB), bottom right of body (BRB), bottom left (BotL), bottom (Bot), and bottom right (BotR). Gaze at the head is defined as gaze at ROI head, and gaze at the robot body is congruent with ROI body, whereas the gaze at any remaining ROIs is coded as ROI gaze averted. These individual ROIs constituting the ROI gaze averted can further be grouped to distinguish between fixations on top and bottom. Gaze fixations that do not fall into any of the defined ROIs are coded with no ROI. These fixations have been added to the general gaze averted ROI but have not been used when splitting gaze aversions into top and bottom. The relevant time span of interaction started at the end of the greeting utterance of the robot up to the start of the farewell message or the utterance of the invitation to speak more.

### 5.2 Power analysis

G*Power (Faul et al., 2009) was used to compute the power of the study design and the compromise of *α* and power. For a sufficiently sized sample, the preferred test statistic would be the one-way ANOVA, as normal distribution is expected in the dependent variables. Therefore, we computed the *a priori* sample sizes using Cohen’s f as effect size (*f* = 0.1, small; *f* = 0.25, medium; and *f* = 0.4, large). With *α* = 0.05, power (1 − *β*) = 0.8, and five groups, the required sample sizes are *n* = 80 for a large effect and *n* = 200 for a medium effect. The actual sample size of *n* = 100 is suitable for an effect size of *f* = 0.355.

The used *η* ^2^ effect size can be transformed into *f* using $f=\sqrt{\eta^{2}/\left(1-\eta^{2}\right)}$ (Cohen, 2013), which results in *η* ^2^ = 0.0828, *f* = 0.3 for the sensible item in the self-reported data.

A compromise power analysis of *q* = *β*/*α* = 4 (as *α* = 0.5, and test power 1 − *β* = 0.8), *N* = 100, and five groups resulted in a compromise value of *α* = 0.10 and test power of (1 − *β*) = 0.60. With this insight, the hypothesis test results that fall into the range of 0.05 < *α* < 0.1 can serve as indicators for future studies (e.g., the capability scale in the self-reported data analysis).

### 5.3 Effect of gender

We did a preliminary check on the effect of gender, as gender has been reported to affect eye gaze behavior Gomez et al. (2019). Because the *t*-test assumptions for a *t*-test between attention, comfort, capability, duration, and word count as dependent variables and gender (f/m) as an independent variable were not met, we performed a Mann–Whitney *U* test on these dependent variables without any significant results. The same applies to the main dependent eye gaze variables, namely, the normalized summed-up fixation durations of the ROIs head, body, and gaze averted. These results, together with the gender stratification in the GAR conditions, lead us to exclude gender as a covariate from further analysis.

### 5.4 “Was that all?”: Robot asked for more information

Among the 96 participants, the robot asked 44 to continue talking about their chosen movie because otherwise, the interaction duration would have been shorter than 2 min. The other 52 participants were not asked to elaborate further. This does not influence the eye gaze measures as the gaze behavior is only evaluated for the interval between the end of the robot greeting utterance and the next robot utterance after that, that is, query for more information or farewell. However, the participants filled in the questionnaire at the end of the interaction. Thus, the possibility that the additional question posed by the robot influenced the perception of the participants must be investigated.

We tested for significant group differences for the attitudinal and behavioral measures using a Kruskal–Wallis test (Supplementary Tables S1, S2). There were no significant differences in the self-report data. Thus, we conclude that the additional question by the robot did not influence the attitudinal measures. However, as expected, there were significant differences in the interaction duration and word count. Participants, who were queried for more information, talked for a shorter amount of time.

### 5.5 Human gaze data evaluation

The 88 participants with valid eye-tracking produced 23,529 fixations. The average fixation count per participant was 247 (SD = 158). The average fixation duration per participant was mean = 404 ms (SD = 215 ms). In total, 913 fixations were categorized as no ROI. The descriptive statistics per GAR condition are shown in Supplementary Table S3.

#### 5.5.1 Fixation duration

We investigate whether the fixation durations differ between the groups per ROI when aggregated over the whole interaction. We normalized the fixation duration sums per participant and averaged them per condition for the three ROIs head, body, and gaze averted between the GAR conditions.

The ANOVA test assumptions are met for the gaze duration at the head but not for the body and the averted ROI.^5^

We performed an ANOVA on the head ROI (Supplementary Table S4) between GAR conditions (*F* (4) = 2.704, *p* = 0.036, *η* ^2^ = 0.117). We performed a Kruskal–Wallis test on the ROI body (*χ* ^2^ (4) = 6.4074, *p* = 0.1707, *η* ^2^ = 0.0294) and the ROI gaze averted (*χ* ^2^ (4) = 3.15, *p* = 0.533, *η* ^2^ = −0.0104). These results indicate that the GAR affects ROI head, not body and gaze averted. However, a Tukey HSD *post hoc* test for pairwise differences in the fixation duration of the head ROI revealed no significant group differences. The lowest adjusted *p*-value occurred between the two groups GAR 0.3 and 0.9 (*p* = 0.065).

**FIGURE 4:**
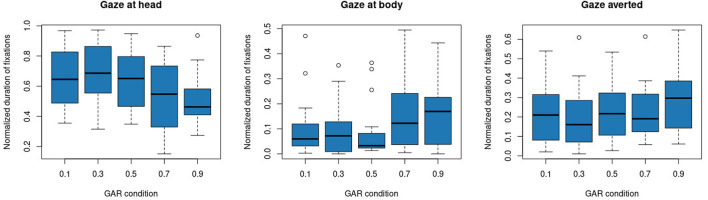
Normalized gaze duration on ROIs, head, body, gaze averted, for each GAR condition.

**FIGURE 5:**
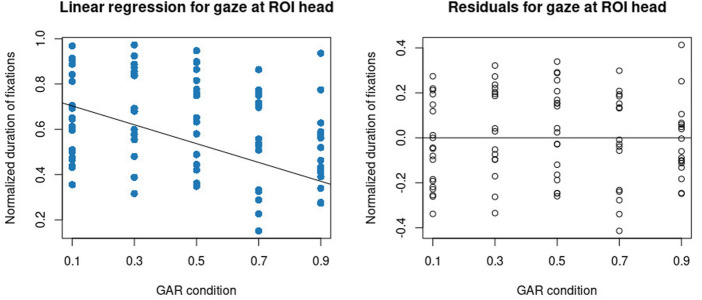
Left: linear regression model between GAR and fixation duration at ROI head. Right: the residuals of the linear regression.

Figure 4 motivated a correlation and linear regression analysis between GAR conditions and fixation duration on the ROI head. GAR is an interval scale regarding its implementation on the robot. We performed a Pearson correlation (*r* = −0.2981547, *t* (85) = −2.8798, *p* = 0.005). The linear regression resulted in an intercept of 0.71 (Std. Err = 0.04, *t* = 16.98, *p* < 0.000) and slope (i.e., the effect of the condition) of −0.21 (Std. Err = 0.07, *t* = −2.88, *p* = 0.005). The multiple R-squared value of the linear regression is 0.089. Visual inspection of the residuals (Figure 5) does not suggest higher-order functional relations between GAR and fixation duration at ROI head, and a quadratic regression analysis was non-significant.

The regression and correlation indicate that the lower the robot’s GAR (i.e., the more the robot gazes at the human), the more the human gazes at the face of the robot. Conversely, the more the robot gazes away from the human, the more the human gazes at the body of the robot.

#### 5.5.2 Fixation duration: Temporal split

Until now, we inspected gaze metrics that are temporally aggregated across the whole interaction duration. In this section, we analyze the influence of time on gaze behavior in the different GAR conditions. Therefore, we split each individual interaction into thirds, similar to Baxter et al. (2014) and Kennedy et al. (2015).

**FIGURE 6:**
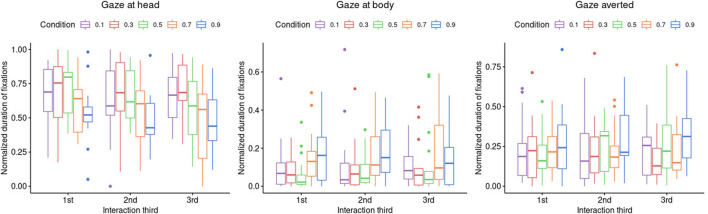
Normalized fixation durations for variable ROIs, head (left), body (middle), and gaze averted (right), for the between-factor *GAR* condition and the within-factor interaction third.

For each third, the gaze metrics are calculated. The ANOVA assumptions are met for the ROI head but not for the other two ROIs, body and gaze averted. Thus, we performed a MANOVA on the ROI head with the within-factor time and the between-factor condition (Table 2; Figure 6), which revealed significant main effects and a significant interaction effect, all with a small effect size.

**TABLE 2:** Top: MANOVA for the within-factor time and the between-factor condition (*α* =0.05 (*)). Middle: ANOVA with the factor condition on the normalized fixation duration of the ROI head for each interaction third. Bottom: ANOVA with the factor time on the normalized fixation duration of the ROI head for each condition.

	F	df1	df2	*p*	*p* adj	*η* ^2^
Condition	2.54	4	80	0.046*		0.093
Time	4.64	2	160	0.011*		0.011
Interaction	2.13	8	160	0.035*		0.020
Time	F	df1	df2	*p*	*p* adj	*η* ^2^
1st third	1.98	4	82	0.106	0.110	0.088
2nd third	2.42	4	82	0.055	0.112	0.105
3rd third	3.38	4	80	0.013	0.039*	0.145
Condition	F	df1	df2	*p*	*p* adj	*η* ^2^
0.1	0.512	2	36	0.604	1.000	0.007
0.3	0.985	2	28	0.386	1.000	0.016
0.5	12.781	2	34	<0.000	<0.000*	0.070
0.7	4.424	2	30	0.021	0.084	0.043
0.9	0.348	2	23	1.000	1.000	0.017

To determine where the interaction effect lies, we performed two groups of one-way ANOVAs: first, we examined the effect of the condition in the separate interaction thirds (middle of Table 2). The condition has a significant and large effect in the last interaction third.

Second, we examined the effect of time in the different conditions *via* another group of ANOVAs (bottom of Table 2). There was a significant difference in the time spent gazing at the robot head (medium effect) between the interaction thirds in the GAR condition 0.5. This finding emphasizes the visual result of Figure 6, where the gaze at the head in the 0.5 GAR condition occurs for a longer duration in the first third than in the other two-thirds. Summarizing the interaction effect, an inspection of the median values in Figure 6 suggests that the gaze durations form a “∧” shape in the first third, whereas this is transformed into a downward slope in the last third over time.

Finally, for the ROI head, Tukey HSD *post hoc* tests for the interaction effect revealed no significant comparisons. However, this can be explained by the unusually high number of group comparisons when both interaction third and condition are examined. For the other two ROIs, body and gaze averted, the ANOVA assumptions were not met. Therefore, the multivariate non-parametric Scheirer–Ray–Hare test was chosen and performed on the ROI body (GAR condition: H (4) = 16.4, *p* = 0.002, time: H (2) = 0.55, *p* = 0.76, interaction H (8) = 3.60, *p* = 0.89) (Figure 6). Only the GAR condition had a significant effect. Dunn *post hoc* tests revealed the two different GAR groups (GAR 0.3, 0.5 and GAR 0.7, 0.9), with the second group exhibiting a higher amount of gazing at the body. GAR 0.1 almost met the significance level for being significantly different from GAR 0.7 and 0.9. The significant group comparisons are 0.1–0.7 (adjusted *p* (*p* adj.) = 0.06); 0.1–0.9 (*p* adj. = 0.06); 0.3–0.7 (*p* adj. = 0.03); 0.3–0.9 (*p* adj. = 0.02); 0.5–0.7 (*p* adj. = 0.02); and 0.5–0.9 (*p* adj. = 0.01). This reinforces the linear regression result of the ROI head: people looked at the robot’s body and not at its face more often when the robot displayed a high GAR.

The procedure was repeated for ROI gaze averted (GAR condition: H (4) = 8.79, *p* = 0.06, time: H (2) = 0.50, *p* = 0.77, interaction H (8) = 2.98, *p* = 0.93) (see Figure 6) without any significant effects.

#### 5.5.3 Analysis of gaze sequences

Similar to Yu et al. (2012) and Broz et al. (2012, 2013), we are interested in whether the different GAR conditions lead to different dynamic gaze patterns in the participants. The gaze directions of the human participants are categorized into ROIs (Figure 3). Thus, every participant produced a sequence of fixations on the ROIs, similar to Lehmann et al. (2017) and Acarturk et al. (2021). For this section, we group the ROIs into the following gaze directions (Figure 3): head (*H* = {*H*}), body (*B* = {*B*}), up (*U* = {*TH*}), up-left (*UL* = {*TLH*, *LH*}), down-left (*DL* = {*TLB*, *BLB*}), up-right (*UR* = {*TRH*, *RH*}), down-right (*DR* = {*TRB*, *BRB*}), and down (*D* = {*BotL*, *Bot*, *BotR*}). Therefore, each participant *i*, (*i* ∈ {1, *…*, *N*}) produces a sequence $S_{i}=\left\{S_{i1},S_{i2}\ldots ,S_{iT_{i}}\right\}$ , with *S*_*it*_ ∈ *D* = {*H*, *B*, *U*, *UL*, *DL*, *UR*, *DR*, *D*} and *t* ∈ {1, *…*, *T*_*i*_}, with *T*_*i*_ as the length of the fixation sequence of participant *i*. For each participant, a sequence of fixation transitions is constructed. They can be used to create a stochastic model of the gaze behavior of a single participant in the form of a discrete-time Markov chain (DTMC) (Papoulis, 1984). For these models, the Markov property assumption dictates that the gaze direction probability depends only on the previous gaze direction. This assumption is expressed as

$$\mathrm{Pr}\left(S_{t+1}=s_{t+1}|S_{1}=s_{1},S_{2}=s_{2},\ldots ,S_{t}=s_{t}\right)=\mathrm{Pr}\left(S_{t+1}=s_{t+1}|S_{t}=s_{t}\right),$$

where the single fixation directions are random variables with the domain *D*. Thus *s*_*t*_ ∈ *D*. Additionally, assuming time-invariance across an interaction, the stationary distribution is represented as a |*D*|×|*D*| transition matrix *M*, where an element *m*_*ij*_, *i*, *j* ∈ {1, *…*, |*D*|}, describes the probability of transitioning from direction *i* to direction *j*, with *∑*_*j*∈{1,*…*,|*D*|}_ *m*_*ij*_ = 1 for all *i* ∈ {1, *…*, |*D*|}. Transitions include loops, which result from starting and ending in the same ROI. This can occur when a participant has two consecutive fixations in the same ROI.

Subsequently, the transition matrix for each participant is created. In order to aggregate them, the single-participant transition matrices are summed up and normalized row-wise. This way, the gaze behavior of each participant influences the outcome with the same weighting, independent of the interaction duration.

For each GAR condition, there is a different DTMC model. Each DTMC is a 14 × 14 transition matrix. Visual inspection of the differences between these matrices can reveal how the gaze transitions differ. However, to determine whether the models differ from each other in a statistically significant way, each of the five 14 × 14 matrices is flattened into a vector of length 196. Then, these five vectors can be stacked into a 5 × 196 matrix. In this representation, each column describes one fixation transition (e.g., head → body). Considering the non-normalized fixation counts, each row describes the categorical distribution of fixation shifts. This matrix is very sparse, containing many zero values for unlikely transitions. To interpret the results and perform a *χ* ^2^ test of independence, we aggregated the fixation shifts into the transitions between the previously defined ROI regions head, body, and gaze averted. This transformation yields a 5 × 9 matrix, with all cell values >5 (Table 3).

**TABLE 3:** Top: observed transition counts between the ROIs body (b), head (h), and gaze averted (a). Bottom: differences in transition counts (observed−expected) for the transitions between the ROIs body (b), head (h), and gaze averted (a) per GAR condition. The adjusted *α* level is 0.00 $\dot{1}$ (*), 0.000 $\dot{2}$ (**), and $0.0000\dot{2}$ (***), respectively. Colored cells highlight comparisons made in Section 5.

GAR	b-b	b-h	b-a	h-b	h-h	h-a	a-b	a-h	a-a
0.1	205	153	67	160	1,870	313	56	324	488
0.3	225	161	79	161	1,517	380	77	374	585
0.5	249	150	67	144	2,298	537	77	530	1,116
0.7	481	248	131	268	1,322	394	103	409	749
0.9	510	267	133	267	1,263	401	134	378	941
0.1	−87 ^***^	−18	−17	−15	422 ^***^	−42	−22	−29	−191 ^***^
0.3	−61 ^**^	−7	−3	−10	99 ^**^	33	0	29	−80 ^**^
0.5	−167 ^***^	−94 ^***^	−52 ^***^	−105 ^***^	239 ^***^	33	−34 ^**^	28	150 ^***^
0.7	151 ^***^	54 ^***^	37 ^***^	70 ^***^	−313 ^***^	−6	15	11	−18
0.9	165 ^***^	65 ^***^	34 ^**^	60 ^***^	−447 ^***^	−18	42 ^***^	−39	139 ^***^

**FIGURE 7:**
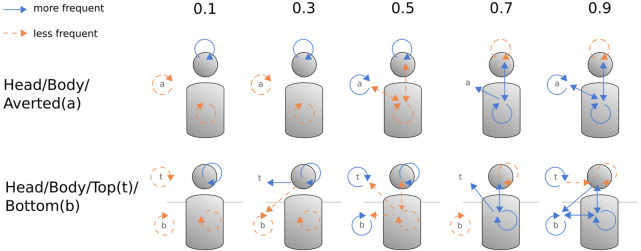
Visualization of significant deviations from the expected gaze transition frequencies in the 5 GAR conditions. Top: ROIs head, body, gaze averted. Bottom: ROIs head, body, top, and bottom.

For this matrix, we performed a *χ* ^2^ test of independence (*χ* ^2^(32) = 973.57, *p* < 2.2*e* − 16, Cramer’s V = 0.108 (weak effect)). The z-scores for the cells were calculated to determine which cells lead to this significant result (where *z* < − 1.96 or *z* > 1.96 would represent a significant deviation without Type I error correction). Then, the *χ* ^2^ values were calculated using the z-scores. For each of the *χ* ^2^ values, the *p*-value was compared against a *χ* ^2^ distribution (df = 1, one-sided) (Beasley and Schumacker, 1995; Garcia-Perez and Nunez-Anton, 2003). The adjusted significance level $\alpha =0.5/\left(5\cdot 9\right)=0.00\dot{1}$ was used to check which cells differ significantly from the expected distribution (Table 3). For example, all entries in the body-to-body transitions column are significant. Negative cell entries indicate that fewer gaze transitions than expected have occurred, whereas positive cell entries indicate that more transitions than expected occurred. Figure 7 visualizes all significant deviations from expected gaze transition frequencies. In Figure 7, solid blue arrows indicate a significant positive result (i.e., more transitions than expected), whereas orange dashed lines indicate significant negative results (i.e., fewer transitions than expected). This graphic only indicates significant deviations from expectations, not the absolute number of transitions. For example, a comparison of the two columns *h-b* and *h-a* (marked in red color) in Table 3 reveals that *h-a* transitions occurred more frequently than *h-b* transitions among all groups. This means, in general, after gazing at the head, participants averted their gaze rather than look at the robot’s body.

However, we are interested in the differences between the GAR conditions, and thus we proceed to cluster the significant cell entries in Table 3 to interpret them on a higher level.

The three self-loop columns in Table 3 for *h-h*, *b-b*, and *a-a* (cells colored in blue) confirm the findings of the gaze behavior with respect to the total time spent gazing at a particular ROI. All entries in column *h-h* display significant differences from the expected occurrences, namely, a higher count for the low GAR conditions 0.1, 0.3, and 0.5 and a lower-than-expected count for the conditions 0.7 and 0.9. Comparable results are visible in columns *b-b* and *a-a*, where the relationship is reversed for the GAR conditions. This means that when the robot gazed at the participants for a longer time, the participants reciprocated the robot’s mutual gaze.

Regarding true fixation shifts (i.e., transitions between different ROIs), a large pattern of significant results can be observed in the submatrix GAR 0.5, 0.7, 0.9 and transitions *b-h*, *h-b*, *b-a*, and *a-b* (cells colored in orange). These transitions occur less frequently for the 0.5 GAR condition and more frequently for the GAR conditions 0.7 and 0.9. This means that in the 0.5 GAR condition, participants shifted their gaze less frequently between the body and the head and less frequently between the body and the gaze averted ROI (although in this GAR condition, gazing at the head was more frequent). The relationship is reversed for conditions 0.7 and 0.9: much fixation shifting between head and body, with less frequent gazing at the head. Similarly, there are more transitions between the body and gaze averted ROI. This means the gaze pattern in the 0.7 and 0.9 conditions centers more around the robot’s body. However, in the 0.5 condition, the gaze at the body occurs less frequently. In short, in the 0.5 GAR condition, significantly fewer fixation shifts occurred between different ROIs, whereas in conditions 0.7 and 0.9, significantly more fixation shifts occurred between different ROIs.

The gaze transition *a-b* happened less frequently in the 0.5 condition and more frequently in the 0.9 condition. Interestingly, there are no differences in the gaze shifts *h-a* and *a-h*. This means there is no difference between the GAR conditions for looking at the robot’s head after averting the gaze. However, this is the gaze shift the robot performed.

In summary, these results confirm that robotic GAR influences human gaze behavior, but not in a direct way. Otherwise, the frequency deviations in the gaze shifts between the ROIs head and gaze averted would be significant as the robot gaze aversion pattern consists solely of shifts between the ROIs head and gaze averted. Participants reacted with more gaze aversion to a high gaze aversion pattern of the robot. However, the participants seemed not to perform mere gaze following. Deviations from the expected frequencies occurred in the gaze pattern centered around the robot’s body to shift the gaze to the robot’s head or averting the gaze.

Gaze aversions in different directions have been associated with different conversational goals. Andrist et al. (2014) reported that people in conversations more often avert their gaze upward when they experience a high cognitive load, and they gaze downward when they need to regulate the level of intimacy. Therefore, we split the single ROIs of the aggregated ROI gaze averted into top (t) and bottom (bot). The ROI top consists of all ROIs above body, except the ROI head. The ROI bottom consists of all ROIs below the head except body (Figure 7). The *χ* ^2^ tests of independence were significant (*χ* ^2^ (60) = 1122.2, *p* < 2.2*e* − 16, Cramer’s V = 0.116 (weak effect)). The results for single significant cells are shown in Table 4. We adjusted the *α* level for top–bottom to *α* = 0.5/(5*12) = 0.0083.

**TABLE 4:** Top: observed transition counts between the ROIs body (b), head (h), top (t), and bottom (bot). Bottom: differences in transition count (observed−expected) for the transitions between the ROIs body (b), head (h), top (t), and bottom (bot). The adjusted *α* level is 0.00083(*), 0.00017(**), and 0.000017(***), respectively. The transitions *h-h*, *h-b*, *b-h*, and *b-b* are omitted, as they are already depicted in Table 3. Colored cells highlight comparisons made in Section 5.

GAR	b-t	b-bot	h-t	h-bot	t-b	t-h	t-t	t-bot	bot-b	bot-h	bot-t	bot-bot
0.1	22	45	254	59	19	263	369	24	37	61	30	65
0.3	27	52	353	27	35	336	454	27	42	38	24	80
0.5	17	50	467	70	23	447	824	41	54	83	27	224
0.7	56	75	343	51	38	346	595	39	65	63	36	79
0.9	35	98	318	83	41	294	688	28	93	84	25	200
0.1	−5	−11	−50	8	−8	−32	−144 ^***^	−4	−14	3	5	−48 ^***^
0.3	0	−3	56^*^	−23^*^	8	47	−48	−0	−8	−18	−0	−31
0.5	−22^**^	−30^**^	35	−2	−16	27	95 ^***^	1	−18	1	−8	63 ^***^
0.7	25^***^	12	−0	−6	7	13	16	8	7	−2	8	−49 ^***^
0.9	3	32 ^***^	−41	23 ^*^	9	−55 ^*^	82 ^**^	−5	33 ^***^	16	−4	66 ^***^

Concerning the general gaze pattern for all groups, the head-to-top, top-to-head, and top-to-top gaze shifts were far more numerous than the gaze shifts regarding the ROI bottom and body (cells marked in blue). This gaze aversion pattern rather indicates a cognitive effort than intimacy regulation. Checking the top to bottom and body-to-bottom columns reveals that this cannot be a measurement artifact. Assuming that the true gaze shift occurred between head and bottom, but the eye tracker falsely registered an intermittent fixation on top or body, we would expect more shifts from the top-to-bottom or body-to-bottom column. However, the frequencies in both columns are relatively low, too.

As mentioned above, we are interested in group differences between the GAR conditions and try to find an explanatory pattern for the significant cells. The following significant results were observed: participants in the 0.5 and 0.9 conditions had more fixation shifts from bottom to bottom. In contrast, in conditions 0.1 and 0.7, this occurred less frequently (cells marked in red). The top-to-top transitions occurred less frequently in the 0.1 condition and more frequently in the 0.5 and 0.9 conditions (cells marked in orange). There were no other significant results for the 0.1 condition. Participants in condition 0.3 made more gaze shifts from head to top than from head to bottom. In condition 0.5, participants gazed less frequently from body to top and from body to bottom. In condition 0.7, more gaze shifts occurred from body to top. There were more significant results in condition 0.9: there were more occurrences of shifts from body to bottom, head to bottom, and bottom to body and fewer occurrences of top to head (cells colored in blue).

Top-to-bottom or bottom-to-top fixation shift frequencies did not differ between groups. Looking for a pattern reveals significant results in the bot—bot column, largely congruent with the *a-a* gaze shift in the previous ROI split. For condition GAR 0.9, the gaze shifts paint a picture of higher frequency gazing downward, indicating intimacy regulation (Andrist et al., 2014) in contrast to the other groups. In summary, splitting the gaze averted ROI into top and bottom reproduces the previous result.

#### 5.5.4 Temporal correlation of robot and human gaze shifts

**FIGURE 8:**
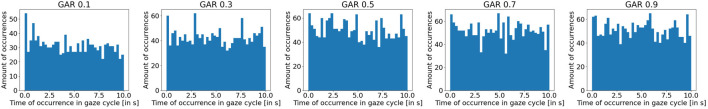
Distributions of the occurrence of a fixation shift of the participants with respect to the 10 s gaze cycle of the robot. From left to right: GAR 0.1, 0.3, 0.5, 0.7, 0.9.

Subsequently, we wanted to examine whether the gaze behavior of the robot causes temporally aligned fixation shifts in the participants. Therefore, for each participant, we calculated the point in time of each real fixation shift (i.e., no loop-like head-to-head) with respect to the 10 s gaze cycle time of the robot. Thus, each fixation has a starting time of 0–10 s with respect to the gaze cycle duration. These starting times per participant are gathered per GAR condition. This results in five distributions, which we tested for significant differences. Because the variables were not normally distributed, we conducted one Kruskal–Wallis test counting all real fixation shifts (*χ* ^2^ (4) = 5.36, *p* = 0.25) and one Kruskal–Wallis test that counted only fixation shifts from and to the head (*χ* ^2^ (4) = 4.31, *p* = 0.36). Thus, there is no evidence for differences in the temporal occurrence of gaze shifts within the 10 s robot gaze cycle. The distribution for all GAR conditions is depicted in Figure 8. The histograms suggest a uniform distribution within the 10 s cycle for all five conditions. This result indicates that the robot gaze shifts do not trigger human gaze shifts. In the previous sections, differences in the gaze behavior between the GAR conditions were found, but the differences cannot be explained by mere gaze following.

### 5.6 Interaction behavior evaluation

**FIGURE 9:**
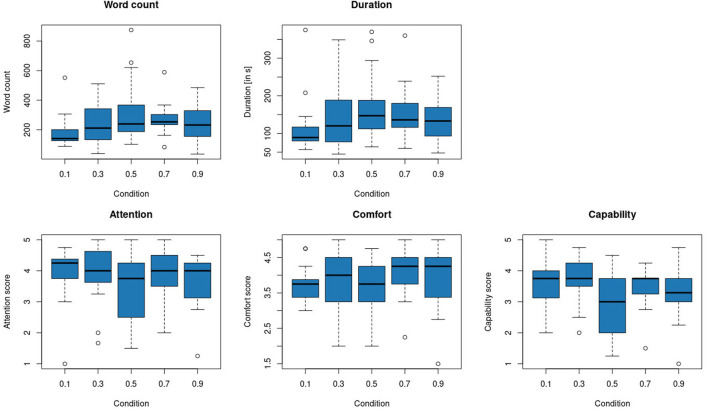
Top: word count (left) and duration (right) per GAR condition. Bottom: attention displayed by the robot (left), comfort during the interaction (middle), and perceived robot capability (right) per GAR condition.

The two variables, *duration* and *word count* (Table 5), were naturally highly correlated (*ρ* = 0.922, *t* = 23.139, *p* < 0.000). The ANOVA assumptions for the effect of GAR on these two variables were not met. Therefore, we applied a Kruskal–Wallis test (Figure 9). The null hypothesis of RQ 2a that there is no effect of the robot’s GAR on the number of words spoken by the participants can be rejected (*χ* ^2^ (4) = 11.3, *p* = 0.02, *η* ^2^ = 0.08). The null hypothesis of RQ 2b that there is no effect of the robot’s GAR on the interaction duration can be rejected (*χ* ^2^ (4) = 11.6, *p* = 0.02, *η* ^2^ = 0.08). For both significant tests, there is moderate effect size.

**TABLE 5:** Descriptive statistics of the word count and interaction duration, as well as the Likert scale *comfort*.

	Word count			Duration			Attention		Comfort		Capability	
Condition	Mean	Median	SD	Mean	Median	SD	Mean	SD	Mean	SD	Mean	SD
0.1	180	140	106	113	89	72	3.92	0.85	3.70	0.50	3.59	0.72
0.3	232	211	139	138	120	80	3.94	0.90	3.76	0.81	3.67	0.69
0.5	309	239	197	168	147	82	3.47	1.15	3.74	0.65	2.94	0.93
0.7	272	253	107	157	136	66	3.84	0.82	4.04	0.66	3.44	0.65
0.9	243	232	120	137	133	55	3.70	0.81	3.89	0.87	3.34	0.81

We conducted pairwise comparisons for word count and duration using the Dunn test with Bonferroni–Holm correction. There are significant differences in the word count and duration between groups 0.1 and 0.5 (*p* adj = 0.03, *p* adj = 0.02), as well as 0.1 and 0.7 (*p* adj. = 0.03 *p* adj. = 0.04).

This means participants in the GAR 0.1 condition (the robot mostly stares at the participant) spoke fewer words and for a shorter amount of time than those in the 0.5 (robot averted gaze half of the time) and 0.7 GAR conditions. Thus, there is a moderately sized effect of the GAR on the interaction level. In conversational settings, listeners tend to gaze at the speaker more often than the speaker gazes at the listener (Argyle et al., 1994). However, we observe that in the 0.1 GAR condition, participants talked significantly less. A possible explanation is that a too low GAR is perhaps not (only) interpreted as benevolently paying attention to the speaker. Together with the gaze behavior results, we observe now that participants in lower GAR conditions (i.e., more robot staring) talked less while gazing more at the robot.

### 5.7 Attitudinal data evaluation

Until now, only behavioral data were evaluated, revealing differences in gaze behavior and interaction duration between the GAR groups. In addition to this, self-reported attitudinal data were recorded to learn more about how participants experienced the conversational situation. The preconditions for ANOVA were not met. We performed Kruskal–Wallis tests to evaluate the research questions about the impact of the GAR conditions on the aggregated scales attention displayed by the robot, comfort elicited by the robot, and robot capabilities (Supplementary Table S5). The mean and standard deviations of the aggregated scales per condition are shown in Table 5 and Figure 9.

There are no significant differences between the GAR conditions with respect to the scales of attention, comfort, and capability (Supplementary Table S5). If there is a meaningful effect, it cannot be detected because the sample size or the actual effect size is too small. Otherwise, the GAR could not have a significant effect on attitudinal measures. Therefore, although the GAR has a moderately sized effect on the behavior level, both for gaze behavior and interaction duration, the participants did not form significantly diverging opinions about the robot in the different conditions.

Additional exploratory evaluations revealed correlations between measures. The Pearson correlation coefficient *r* was calculated for the three composite self-reported scales, attention, comfort, and capability, on the one hand and the behavioral data, word count and duration, on the other (Table 6). When aggregated for the whole dataset, no extreme outliers were detected in these variables. The data suggest a moderate negative correlation between the participants’ perception of attention shown by the robot and interaction duration and word count. The longer the interaction took, the less the participants felt the robot was paying attention to them. A possible reason is the missing backchannel communication (e.g., utterances like “mhm” and “aha”) or gestures (e.g., nodding) that humans use during prolonged periods of listening.

**TABLE 6:** Pearson correlations between self-reported (attention, comfort, and capability) and behavioral measures (duration and word count) and pupil size (avg. pupil size and pupil size SD) for the whole sample.

	Attention			Comfort			Capability		
	t	p	r	t	p	r	t	p	r
Duration	−2.81	0.005**	−0.27	−0.47	0.63	−0.04	−2.00	0.04*	−0.20
Word count	2.72	0.007**	−0.27	−0.18	0.85	−0.01	−2.20	0.02*	−0.22
Avg. pupil size (mm)	2.22	0.02*	0.23	−0.27	0.78	−0.02	−0.47	0.63	−0.05
Pupil size SD (mm)	1.56	0.12	0.16	1.97	0.05*	0.21	−0.33	0.73	−0.03

Correlations for the attitudinal measures and the physiological measures, average pupil size and pupil size variance, revealed a significant moderate positive correlation between the pupil size and the attention score (Table 6). There is also a significant moderate positive correlation between pupil size variance and the level of comfort. These findings affirm previous psychophysiological studies: Beatty (1982) and Joshi et al. (2016) found that cognitive processes are associated with constantly higher pupil dilations. One can speculate that participants who put more effort into retelling a movie exhibited more cognitive effort, which led to a larger pupil dilation and a higher self-worth protecting assignment of the robot’s attention to their story. Similarly, participants who might have felt a certain comfort in the interaction thus exhibited a higher pupil dilation variance. This is in accordance with Partala and Surakka (2003), where pupil dilation variance is positively associated with affective processing. If this is the case in this experiment, to arrive at the positive correlation between pupil variance and comfort attribution, the elicited affect spectrum ranges from neutral to positive.

## 6 Discussion

In this section, we will discuss and summarize 1) the findings regarding the three research questions, 2) the limitations of the study, and 3) the design recommendations resulting from our work.

### 6.1 Answering the research questions

The previous section presented the statistical test results of the research questions. For the three sub-questions (RQ 1a–c) on gaze behavior, several different analytical methods were used because of the wealth of data from the eye-tracking procedure. There are fewer metrics for the two sub-questions (RQ 2a and b) on interaction behavior and the three sub-questions (RQ 3a–c). They will be used to corroborate the gaze tracking results.

#### 6.1.1 RQ 1: Gaze behavior

For all three sub-questions of RQ 1, the null hypothesis can be rejected: GAR does affect the fixation durations (RQ 1a). The effect of GAR on the fixation durations changes during the interaction (RQ 1b). The GAR does influence the fixation sequence behavior of the participants (RQ 1c).

The analysis of the fixation shifts was structured to advance from overall differences of fixation durations on large ROIs, head, body, and gaze averted, to fine-grained dynamic gaze shift patterns.

The main point of discussion is whether the robotic gaze behavior influences the gaze behavior of the participants and the attitudes of the participants toward the robot. We use gaze as a proxy measure of attitudes toward the robot by incorporating correlations between gaze behavior and affect presented in the related work. These results are compared with the self-reported attitudes and the interaction duration, another proxy measure of engagement.

There are two competing interpretations regarding the linear regression results across all groups for the overall head GAR behavior between the groups: 1) participants mirror the GAR of the robot (i.e., they create rapport) (St-Yves, 2006) or 2) participants find the interaction with higher GAR more uncomfortable and therefore avert their gaze more (Argyle and Dean, 1965). We discussed another proxy measure from the related work, namely, the interaction length (Sidner et al., 2005): the longer a participant keeps up the conversation, the more engaged they are with the robot, and this again is a sign of comfort during the interaction. Thus, because participants in the 0.1 GAR condition (high robot mutual gaze) talked for a shorter amount of time, this indicates that the rapport explanation is more likely.

However, there is a noteworthy deviation from the linear regression: in the linear regression of the normalized fixation durations for the whole interaction, the 0.1 GAR setting does not adhere to the otherwise linear relationship between the participant and the robot GAR. For a stronger relation, the median of the 0.1 setting was supposed to be higher than that of the 0.3 setting, but this was not the case. This might indicate that the overall trend of participants mirroring the robot’s GAR is invalid at the 0.1 end of the GAR spectrum, where the robot stares at the human. Otherwise, when the robot has a higher GAR and thus averts the gaze more often, participants mirror this behavior instead of increasing the gaze toward the robot. If we suppose the robot was perceived as a social agent and creating rapport is a typical human adaptation in a conversation setting, another effect must have been stronger at this end of the robot GAR scale and prevented GAR mirroring. This might indicate that the 0.1 setting of the robot was perceived as uncomfortable, and participants felt being stared at. They sought to regulate the intimacy level by avoiding their gaze more often. This indicates that high mutual gaze is not always a justified proxy measure of comfort in the interaction, as suggested by Castellano et al. (2010), Papadopoulos et al. (2016), Baxter et al. (2014), and Jokinen (2018).

Subsequently, we studied how the fixation distribution changes over the duration of the interaction. Similar to Perugia et al. (2021) and Jokinen (2018), we also observed a time effect. In their study, the gaze toward the robot head increased over time, whereas Jokinen (2018) observed a decrease in time spent gazing at the robot head. The second result is replicated in our work. Perugia et al. (2021) argued that later interactions represent a more stable interaction pattern. If this statement was also applicable to single interactions, we can interpret the decreasing gaze at the robot as an effect of habituation or the wearing-off of the novelty effect. Regarding the interaction effects, the GAR condition has the strongest effect in the last interaction third, where we can observe a linear regression. However, for the linear regression of the whole interaction, the median of the 0.1 GAR is not as high as expected. Time as a single factor also showed an effect across all conditions, namely, the decrease in time spent gazing at the head of the robot. The strongest interaction effect of time and GAR occurs in the 0.5 GAR condition. The gaze at the robot’s head is higher in the first third than in the other two thirds.

Then, we were interested in the fixation shifts of the participants. The statistical tests revealed which fixation shifts occurred more or less frequently than expected. The fixation shifts that started and ended in the same ROI largely reproduced the findings of the normalized fixation duration analysis: the true fixation shifts (i.e., when the start and end ROI are not equal) show a pattern of higher gaze aversion for the 0.7 and 0.9 GAR setting, but noticeably a pattern of less gaze aversion in the 0.5 condition. This could indicate a higher comfort for the participants in the 0.5 condition. However, this identified pattern also does not follow the results of the linear regression above.

We tried to distinguish between top and bottom gaze aversion. There, the gaze aversion pattern for the 0.7 and 0.9 GAR conditions indicated an intimacy regulation gaze aversion pattern that typically occurs in uncomfortable situations (Andrist et al., 2014).

Lastly, in this section, we explored the temporal relationship between robot and human gaze shifts, that is, if the human participants engage in gaze behavior after a certain amount of time after a robot gaze shift. This test produced non-significant results and might be another indicator toward the conclusion that humans react to the robotic gaze behavior, but not by the mere gaze following the robot.

#### 6.1.2 RQ 2: Interaction behavior

We can reject the null hypothesis for RQ 2 and thus state that the GAR does affect the number of spoken words (a) and the interaction duration (b). In the related work, interaction duration was used as a proxy measure of engagement (Sidner et al., 2005). Using the same argument, we can conclude that in the 0.1 GAR setting, engagement was the lowest. This, together with the conclusion of the 0.1 linear regression outlier in the above subsection, paints a picture of an uncomfortable situation in the 0.1 setting.

However, this contrasts the exploratory identified significant negative correlation between attention and interaction duration. This might indicate that interaction duration should not be used alone as a proxy measure of interest.

#### 6.1.3 RQ 3: Attitudes

The results for self-reported attitudinal scales perceived attention shown by the robot (RQ 3a), the level of comfort of the participants (RQ 3b), and the perceived robot capability (RQ 3c) did not significantly differ between GAR conditions. We argue against the existence of a medium or large effect of GAR on the conscious perception of the robot interaction in this interaction setting. In the following, we want to contextualize our line of thought.

The median value for attention was highest in the 0.1 GAR setting (though not significantly different from other settings). Together with the shortest interaction duration, this could indicate that participants felt “watched” in a negative sense. The lowest median attention and capability value occurred in the 0.5 GAR group, where there was significantly less gaze-shifting behavior on the body of the robot, though these values were not significantly different.

In the qualitative interview, participants repeatedly mentioned that they experienced the robot GAR as a “turning the ear toward the speaker to better listen.” Most participants mentioned both positive and negative aspects of the interaction, for example, “It was weird in the beginning, then it was nice”; “jerky moves, but cool and nice”; “less weird than expected”; and “funny and strange.” The robot implementation resulted in a range of descriptions, even for the same GAR setting: “The robot seems alive, especially the eye blinking and head movements” on the one hand, and “The robot did not react at all; there was no interaction” on the other hand. Both statements occurred in the 0.1 GAR setting. Many participants mentioned a missing backchannel communication of the robot while listening.

The occurrences of “I don’t know” answers in the attitudinal measures were counted as omissions and indicated what participants can judge with certainty. Participants always knew whether the robot was creepy, they were feeling nervous, or the robot appeared warm, pleasant, or artificial, all with zero omissions. However, they were less certain about whether the robot was ignorant, unconscious, incompetent, or intelligent, all with four-to-six omissions. This might indicate that the type of data collection is inadequate for certain topics. The participants were sure when introspective questions were posed and more unsure when statements about the inner working of the robot were asked about. More specific questions tailored to the interaction might be adequate for such external evaluations.

### 6.2 Limitations

The following limitations and considerations should inform future work. The remarks concern the interaction setting and the statistical evaluation.

Participants were engaged when talking to the robot. Therefore, we consider “talking about a movie” as a suitable main task in our setting. However, some participants mentioned in the interview that the task was stressful, even though they were explicitly told that the interaction was in no way a test.

However, was the simulation of an autonomous robot listening to them convincing for the participants? In accordance with the WoZ reporting guidelines (Riek, 2012), we reported that participants mentioned that they found the robot behavior very sophisticated, or rather basic, as well as very entertaining or boring. Some of them asked about the natural language processing capabilities of the robot before filling out the questionnaire. In this case, the experimenter asked them to wait with the question until after filling out the questionnaire. The degree of awareness about which parts of intelligent behavior are difficult to achieve on a technical basis might have influenced their perception.

However, there was no floor or ceiling effect in attitudinal measures, which indicates that the different parts of the behavior implementation (greeting and farewell procedure, idle behavior, and gaze behavior) were adequate.

We did not attempt to categorize the movie genres of the described movies. Arguably, the chosen genre might have had an emotional priming effect. Remembering the plot of a comedy in contrast to a drama might change the participants’ perception of the robot.

Next, we talked about the limitations of the statistical evaluation. We wanted to establish a functional relationship between GAR in the range of 0.1 and 0.9. However, it is difficult to produce sample points for the continuous range of 0.1–0.9. Therefore, we settled on 0.2 increments between conditions.

As we have observed, the human gaze changes during the interaction. An avenue of gathering more data across multiple GAR parameters would be to change the GAR of the robot continually during the interaction and interpret the associated gaze responses of the participants. This method might produce detailed insights into continuous ranges of GAR.

As we did explore our hypotheses without prior assumptions, comparing all groups to each other could produce arbitrary relationships, as opposed to linear regression. However, *post hoc* group comparisons for even five groups lead to a very strict alpha-level correction. Thus, further increasing the granularity of the independent variable seems infeasible for the empirical topic of our work.

Another difficulty of the granularity of data was the interpretation of gaze sequences. With five conditions and three ROIs, the resulting table of statistical test results is only useful for researchers if a pattern can be extracted. However, this allowed different groupings of ROIs to answer different questions (i.e., the split of gaze averted or top/bottom). Additionally, the granularity of gaze sequences is adequate as a basis for learning robot gaze behavior from human data.

Other factors concern the ecological validity of the interaction as the study occurs in a controlled laboratory setting and the relatively homogenous participant sample, namely, persons spending time in the TU Wien library. Populations with different age ranges and cultural backgrounds might give different ratings and show different gaze behavior than the participants of this study.

### 6.3 Design recommendations

In this study, we wanted to determine whether human-inspired gaze settings are the only way to provide agreeable HRI robot behavior parameters. We found that on a conscious level, participants did not distinguish between the different GAR settings, but there were significant behavioral differences.

Designers who aim for long robot interactions with users should aim for a higher GAR setting (i.e., avert the gaze more often).

Designers should be aware that users will start to imitate the gaze behavior of the robot with respect to the overall amount of gazing at different ROIs, which might impact interaction goals.

For robot platforms adhering to the honest anthropomorphism principle (Kaminski et al., 2016), it should be unproblematic to incorporate additional gaze behavior during a conversation with a specific user. For example, in an elderly care home, the robot could have a conversation with one resident while performing fall detection monitoring on its surrounding.

We observed that although the robot gaze behavior was not inspired by human parameters, in some aspects, participants reacted as if the robot was a social agent. Thus, sticking to human-inspired values might be a good starting point for a social behavior implementation. However, when technological aspects demand a deviation from these settings, this does not necessarily lead to a worse perception of the robot by the users.

The research results also warrant considerations in other human-computer interaction settings, such as eye gaze correction in video conferencing software.^6^ Computationally changing the gaze target of human conversation partners might affect the naturally occurring turn-taking behavior of the participants or lead to different interaction durations.

## 7 Conclusion and future work

We conducted an experiment in a controlled laboratory setting where a robot asked participants to talk about their favorite films. While listening to the participants, the robot used different GARs between condition groups, from 0.1 (gazing away only 10% of the time) to 0.9 (averting the gaze to the side 90% of the time). We measured how this factor influenced the UX through eye-tracking, behavioral measures of interaction duration and word count, and self-reported attitudinal data. By varying the independent GAR parameter over a broad range of values that were intentionally not human-inspired, we observed that human participants adjusted their GAR to the robotic GAR, but not by mere gaze following. Participants showed gaze aversion behavior usually associated with uncomfortable interactions when the robot also averted the gaze most of the time. However, the freely chosen interaction duration was the shortest when the robot stared (GAR 0.1) at the participants. As the interaction duration has previously been used as a proxy for comfort in social interaction, this is not an intuitive finding and suggests that adapting the GAR in interaction is a stronger mechanism than gaze aversion to regulate the level of intimacy in an uncomfortable interaction. Regarding the self-reported attitudinal measures, participants did not rate a single GAR setting significantly better or worse than others with respect to the attention or capability of the robot or the comfort in the interaction. However, there are behavioral indicators that the GAR settings on both ends of the parameter range were more uncomfortable than medium parameters. These findings suggest that robot behavior designers can use the robot GAR to influence the interaction duration if necessary, with the caveat that intense robot staring might be uncomfortable. In some situations, robots might have tasks that are concurrent with social interaction (e.g., fall detection and reception work). This might require the robot to avert its gaze to gather information from its surroundings. Our work suggests that in such cases, the robot designers can quite freely adjust the GAR to suit the parallel task, as it does not affect the conscious UX. To summarize, humans seem to apply social gaze behavior toward robots, even if the robot gaze behavior is not implemented by relying on predetermined human gaze parameters.

## Data Availability

The datasets presented in this study can be found in online repositories. The names of the repository/repositories and accession number(s) can be found at: 10.48436/frswc-4dn44.

## Footnotes

1
https://www.softbankrobotics.com/emea/en/pepper.

2
https://www.softbankrobotics.com/emea/en/pepper.

3
https://www.softbankrobotics.com/emea/en/nao.

4 The chosen parameter settings for the fixation classifier are dispersion duration, maximum dispersion = 3°, minimum duration = 100 ms, maximum duration = 4,000 ms, and single marker calibration choreography in Pupil Player v3.3.0.

5 The results of the analysis of the fixation counts yield largely the same results, but the summed fixation duration is more widely used and more principled.

6 NVIDIA Eye Contact: https://www.nvidia.com/en-us/geforce/news/jan-2023-nvidia-broadcast-update/.
