# Supplementary material for: Robotic gaze and human views: A systematic exploration of robotic gaze aversion and its effects on human behaviors and attitudes
Source: Front Robot AI. 2023 Apr 10;10:1062714. doi: 10.3389/frobt.2023.1062714 (PMC10123290; doi:10.3389/frobt.2023.1062714)
Supplement: Supplementary file 1 [file DataSheet2.PDF]

Questionnaire Pepper’s Movies

Participant#:
Condition:
Folder#:
Date, time:

Thank you for participating in our experiment. Please answer some questions for us!
(Questions about Pepper are on the back)

About you

Your age:

Your gender:

Your main profession:

Your previous experience with robots:

never	once	a few times	regularly
◯	◯	◯	◯

Your use of computers:

never	sometimes	daily
◯	◯	◯

Do you wear optical glasses for the experiment (apart from the eye tracker):

yes	no
◯	◯

Can you see the robot clearly:

yes	no
◯	◯

About Pepper:

1. Please rate your impression of **the way you were being attended** by the robot, based on the following statements:

please mark one oval per row

	Strongly disagree				Strongly agree	Don't know
Pepper made a **responsive** impression	◯	◯	◯	◯	◯	◯
Pepper made an **interactive** impression	◯	◯	◯	◯	◯	◯
Pepper made an **ignorant** impression	◯	◯	◯	◯	◯	◯
Pepper made an **unconscious** impression	◯	◯	◯	◯	◯	◯

2. Please rate your **feeling of comfort** with the robot, based on the following statements:

please mark one oval per row

	Strongly disagree				Strongly agree	Don't know
Pepper made a **creepy** impression	◯	◯	◯	◯	◯	◯
Pepper made me **feel nervous**	◯	◯	◯	◯	◯	◯
Pepper made a **warm** impression	◯	◯	◯	◯	◯	◯
Pepper was acting **pleasantly**	◯	◯	◯	◯	◯	◯

3. Please rate your impression of the robot's **interaction capabilities** based on the following statements:

please mark one oval per row

	Strongly disagree				Strongly agree	Don't know
Pepper's interactions were **artificial**	◯	◯	◯	◯	◯	◯
Pepper made an **incompetent** impression	◯	◯	◯	◯	◯	◯
Pepper was acting **intelligently**	◯	◯	◯	◯	◯	◯
Pepper was acting **sensibly**	◯	◯	◯	◯	◯	◯
