# Supplementary material for: Robotic gaze and human views: A systematic exploration of robotic gaze aversion and its effects on human behaviors and attitudes
Source: Front Robot AI. 2023 Apr 10;10:1062714. doi: 10.3389/frobt.2023.1062714 (PMC10123290; doi:10.3389/frobt.2023.1062714)
Supplement: Supplementary file 2 [file DataSheet3.PDF]

Optional glossary (Merriam Webster & Cambridge Dictionary)

responsive
quick to respond or react appropriately or sympathetically

interactive
Mutually or reciprocally active

ignorant
lacking knowledge or comprehension of the thing specified

unconscious
not knowing or perceiving | not aware

creepy
strange or unnatural and making you feel frightened

nervous
tending to produce agitation or nervousness | uneasy

warm
marked by or readily showing affection, gratitude, cordiality, or sympathy

pleasant
enjoyable, attractive, friendly, or easy to like

artificial
lacking in natural, lifelike qualities

incompetent
not having the ability to do something as it should be done

intelligent
able to learn and understand things easily

sensible
based on or acting on good judgment and practical ideas or understanding
