# Supplementary material for: Robotic gaze and human views: A systematic exploration of robotic gaze aversion and its effects on human behaviors and attitudes
Source: Front Robot AI. 2023 Apr 10;10:1062714. doi: 10.3389/frobt.2023.1062714 (PMC10123290; doi:10.3389/frobt.2023.1062714)
Supplement: Supplementary file 3 [file DataSheet1.pdf]

# Supplementary Material

## 1 TABLES

### 1.1 "Was that all?" - Robot asked for more information

| Question asked | Attention mean | Attention SD | Comfort mean | Comfort SD | Capability mean | Capability SD | Word count mean | Word count SD | Duration mean | Duration SD |
|---|---|---|---|---|---|---|---|---|---|---|
| No | 3.62 | 0.953 | 3.87 | 0.768 | 3.28 | 0.912 | 332 | 139 | 185 | 70.9 |
| Yes | 3.94 | 0.852 | 3.77 | 0.632 | 3.51 | 0.630 | 150 | 65.4 | 93.2 | 36.1 |

**Table S1.** Descriptive statistics between the two participant groups "robot asked for more: yes and no".

| | Attention | Comfort | Capability | Word count | Duration |
|---|---|---|---|---|---|
| $\chi^2$ | 3.284 | 1.548 | 0.536 | 52.678 | 53.702 |
| p | 0.069 | 0.213 | 0.464 | <0.000 | <0.000 |

**Table S2.** Kruskal-Wallis tests for differences between the "robot asked for more: yes and no" conditions.

### 1.2 Human Gaze Data Evaluation

| GAR | n | mean (ms) | median (ms) | SD (ms) |
|---|---|---|---|---|
| 0.1 | 3783 | 372 | 219 | 372 |
| 0.3 | 4221 | 384 | 234 | 411 |
| 0.5 | 5799 | 406 | 250 | 453 |
| 0.7 | 4896 | 350 | 219 | 360 |
| 0.9 | 4830 | 330 | 219 | 310 |

**Table S3.** Descriptive statistics for fixations per GAR condition. 0.1: Robot mostly stared at the human. 0.9: Robot mostly looked away from the human. Number of recorded fixations per condition (n), mean, median, and SD of fixation durations per condition.

| GAR | mean | SD | median |
|---|---|---|---|
| 0.1 | 0.65 | 0.19 | 0.65 |
| 0.3 | 0.69 | 0.2 | 0.69 |
| 0.5 | 0.65 | 0.2 | 0.65 |
| 0.7 | 0.54 | 0.22 | 0.55 |
| 0.9 | 0.51 | 0.17 | 0.46 |

**Table S4.** Descriptive statistics for the normalized fixation duration on ROI *head* for all five GAR conditions.

### 1.3 Attitudinal Data Evaluation

## 2 ROBOT SCRIPT

We used the Pepper standard voice in English and the animations from the Pepper's movement library, provided by the NAOqi Python API.

| | $\chi 2$ | p | | $\eta^2$ |
|---|---|---|---|---|
| attention | 2.588 | 0.628 | | −0.015 |
| comfort | 4.685 | 0.321 | | 0.007 |
| capability | 8.689 | 0.069 | · | 0.051 |
| artificial | 2.578 | 0.630 | | −0.015 |
| incompetent | 8.107 | 0.087 | · | 0.045 |
| intelligent | 6.026 | 0.197 | | 0.022 |
| sensible | 11.533 | 0.021 | * | 0.082 |

**Table S5.** Kruskal-Wallis tests for the Likert scales *attention*, *comfort*, and *capability*, as well as the Likert items *artificial*, *incompetent*, *intelligent*, and *sensible*. Effect sizes: $> 0.01$ small effect, $> 0.06$ medium effect and $> 0.14$ (large effect). Significance level: '·': $p < 0.1$, '*': $p < 0.05$.

**Pepper Greeting:**

- Say: "Hello!", Animation Slowly Offer Both Hands
- Say: "My name is Pepper!", Animation Both Hands Bump With Bump
- Say: "Please tell me about a movie you like! What is it about?", Animation Slowly Offer Both Hands

**Pepper Farewell:**

- Say: "Okay! Thanks for speaking to me.!", Animation Slowly Offer Both Hands
- Say: "I think, I now know enough about the movie.", Animation Both Hands Bump With Bump
- Say: "Thanks for your thoughts about it.", Animation Slowly Offer Both Hands
- Say: "Until next time! Bye!", Animation Happy

**Pepper Parameters:**

Speed values fall between 0 and 1, where 1 is specified as the maximum speed. Voice speed: 85, voice shaping: 110, gaze shift speed for pitch: 0.1, gaze shift speed for yaw: 0.2, gaze iteration duration: 10.0, gaze offset: 25, breath amplitude: 0.1, breath bpm: 10, blink duration: 0.05, speed postures: 0.3, speed head tracking: 0.1, head mov max range: 0.02, head mov min range: 0.01, speed speed random head movement: 0.3.

**Pepper Gaze:**

Gaze shifts are programmed to happen within 0.5s. When gazing away, the direction (left/right) is chosen randomly and a target head yaw is then $\pm 25°$. The joint position profiles are smoothly curved [1] and jerky movement is avoided. When directing the gaze back from the averted state to the mutual gaze state, the last known angle position of the face of the human is targeted, until the human face is detected again. Then, the new position is targeted instead. A p-controller scheme is used to avoid jerky movements. While gazing at the human, pepper tracks the face of the human. Random head movement is applied, whenever the robot does not perform gaze shifts.

## 3 PARTICIPANT INSTRUCTIONS

### Written Experiment Introduction

Before signing a written consent form, participants read the following written introduction or the experimenter provided a verbal introduction following the written introduction. This is a short study on

[1] https://developer.softbankrobotics.com/nao6/naoqi-developer-guide/naoqi-apis/naoqi-motion/almotion/joint-control, Case 3: Reactive Control

human-robot conversation. We want to know how people talk to a robot in certain situations to improve the human-robot dialogue. You help us a lot by participating. In this room you see the robot Pepper. Pepper speaks in English. You can talk in English, but if you feel uncomfortable, you can also talk in German. The robot will ask you about a movie you like. Just answer by describing one of the best movies you have watched recently in a couple of minutes and speak about it for about 2 minutes. The robot can see and hear you, just talk like you would normally talk to a friend. Your task is: Just answer by describing one of the best movies you have watched recently in about 2 minutes. Be aware that Pepper will not answer any questions. Pepper's task is: It will simply try to understand you. This study is done to gather data to improve human-robot dialogue, but it doesn't test you in any way. Afterwards we will give you a questionnaire and ask you a few questions about your experience in person.

**Verbal Experiment Introduction** Before manually triggering the start of the experiment, the experimenter elaborated the following points:

- So, this is the robot Pepper. It can hear and see you.
- Pepper will ask you to tell it about a movie.
- Your task is to just answer by describing one of your favorite movies or a movie you watched recently.
- Be aware that pepper will not answer any questions.
- It will simply try to understand you.
